# An advanced bioinformatics approach for analyzing RNA-seq data reveals sigma H-dependent regulation of competence genes in *Listeria monocytogenes*

**DOI:** 10.1186/s12864-016-2432-9

**Published:** 2016-02-16

**Authors:** Yichang Liu, Renato Hohl Orsi, Kathryn Jean Boor, Martin Wiedmann, Veronica Guariglia-Oropeza

**Affiliations:** Department of Food Science, Cornell University, Ithaca, NY 14853 USA

**Keywords:** RNA-seq, *Listeria monocytogenes*, Sigma H, Competence

## Abstract

**Background:**

Alternative σ factors are important transcriptional regulators in bacteria. While σ^B^ has been shown to control a large regulon and play important roles in stress response and virulence in the pathogen *Listeria monocytogenes*, the function of σ^H^ has not yet been well defined in *Listeria*, even though σ^H^ controls a large regulon in the closely related non-pathogenic *Bacillus subtilis*.

**Results:**

Using RNA-seq characterization of a *L. monocytogenes* strain with deletions of all 4 genes encoding alternative σ factors (Δ*BCHL*), which was further modified to overexpress *sigH* (Δ*BCHL::P*_*rha*_*-sigH*), we identified 6 transcription units (TUs) that are transcribed from σ^H^-dependent promoters. Five of these TUs had not been previously identified. Identification of these promoters was facilitated by use of a bio-informatics approach that compared normalized RNA-seq coverage (NRC), between Δ*BCHL::P*_*rha*_*-sigH* and a Δ*BCHL* control, using sliding windows of 51 nt along the whole genome rather than comparing NRC calculated only for whole genes. Interestingly, we found that three operons that encode competence genes (*comGABCDEFG*, *comEABC*, *coiA*) are transcribed from σ^H^-dependent promoters. While these promoters were highly conserved in *L. monocytogenes*, none of them were found in all *Listeria* spp. and *coiA* and its σ^H^-dependent promoter were only found in *L. monocytogenes*.

**Conclusions:**

Our data indicate that a number of *L. monocytogenes* competence genes are regulated by σ^H^. This σ^H^-dependent regulation of competence related genes is conserved in the pathogen *L. monocytogenes*, but not in other non-pathogenic *Listeria* strains. Combined with prior data that indicated a role of σ^H^ in virulence in a mouse model, this suggests a possible novel role of σ^H^-dependent competence genes in *L. monocytogenes* virulence. Development and implementation of a sliding window approach to identify differential transcription using RNA-seq data, not only allowed for identification of σ^H^-dependent promoters, but also provides a general approach for sensitive identification of differentially transcribed promoters and genes, particularly for genes that are transcribed from multiple promoter elements only some of which show differential transcription.

**Electronic supplementary material:**

The online version of this article (doi:10.1186/s12864-016-2432-9) contains supplementary material, which is available to authorized users.

## Background

At the transcriptional level, bacterial gene expression under rapidly changing environmental conditions is controlled by changes in associations between different alternative σ factors and the catalytic core of RNA polymerase. Alternative σ factors are important contributors to gene expression under stress conditions in both Gram-positive and Gram-negative bacteria [1]. In the foodborne pathogen *Listeria monocytogenes*, four alternative sigma factors (σ^B^, σ^H^, σ^L^, σ^C^) play a role in transcriptional regulation. σ^B^ and the σ^B^ regulon have been well defined in this pathogen through a variety of different approaches [2–4]. A number of different studies have indicated that σ^B^ controls a large regulon that plays important roles in both stress response and virulence. The regulons controlled by the other alternative σ factors in *L. monocytogenes* have been less well defined.

While σ^H^ specifically has been shown to play an important role in regulating spore formation and competence in *B. subtilis* [5], the function of σ^H^ in *Listeria* has not yet been well defined. In *B. subtilis*, σ^H^ has been reported to regulate >400 genes (approx. 240 genes positively and approx. 180 negatively) [6]. *B. subtilis* σ^H^ is also involved in the transition from exponential phase to stationary phase, nutrient transport, and the regulation of many other transcription factors and cell-wall-binding proteins [7]. In *L. monocytogenes* characterization of a *sigH* null mutant suggested a role for σ^H^ in growth on minimal medium and under alkaline conditions as well as a role in virulence, as assessed in an intraperitoneal inoculated mouse model [8]. While previous microarray experiments identified 56 genes as being directly upregulated by σ^H^, these experiments also found that the largest category of co-regulated genes were represented by genes that showed transcript levels affected by both the σ^B^ and σ^H^ deletions [9]. Based on this considerable overlap between the σ^B^ and σ^H^ regulon, we surmised that different approaches are needed to further define σ^H^-dependent genes in *L. monocytogenes*. In order to eliminate redundancies between transcriptional regulation by σ^H^ and other alternative σ factors, we chose to perform RNA-seq based comparisons of transcript levels between a *L. monocytogenes* mutant with deletions of all 4 genes encoding alternative σ factors (Δ*BCHL*), and one with the same background that was modified to overexpress *sigH* (Δ*BCHL*::P_rham_-*sigH*). We also implemented an advanced bio-informatics approach where we compare normalized RNA-seq coverage (NRC), between these two strains using a sliding window of 51 nt and 25 nt overlap along the whole genome, rather than comparing NRC calculated only for whole genes; a similar approach was detailed independently in a recent study [10]. We surmised that this approach would allow for more sensitive identification of differentially regulated genes and gene fragments, particularly for genes that may be preceded by multiple promoters (e.g., a σ^H^ and a σ^A^ dependent promoter), where differential transcript levels may only be detectable downstream of the σ^H^-dependent promoter, but not in the actual open reading frame (ORF) where transcription from the σ^A^-dependent promoter may obscure the differential transcription from the σ^H^-dependent promoter.

## Results and discussion

### A sliding window method for identification of differentially regulated genes and promoters provides a sensitive approach for identification of σ^H^ dependent genes

**Fig. 1:**
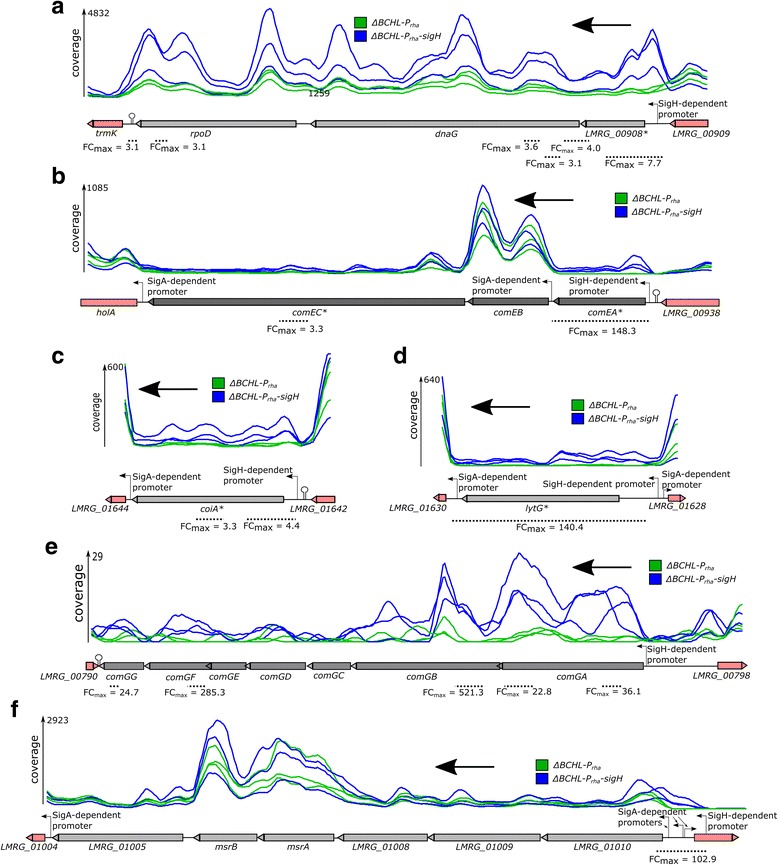
Schematic of σ^H^-dependent transcriptional units identified here. **a** LMRG_00908-*dnaG*-*rpoD* operon; **b** *comEABC* operon; **c** *coiA*, **d** *lytG*; **e** *comGABCDEFG* operon; **f** LMRG_01010-LMRG_01005 operon. *Lines* show average RNAseq coverage for a sliding window of 100 nt. *Blue lines* indicate RNA-seq coverage for the three replicates experiments with the 10403S::Δ*BCHL P*_*rha*_ *-sigH* strain (which over expresses *sigH*), while *green lines* indicate RNA-seq coverage for the three replicates experiments with the 10403S::Δ*BCHL P*_*rha*_ strain (which does not contain *sigH*). Maximum average coverages are shown on the left side of each panel. *Black arrows* indicate the direction in which the RNA-seq reads were mapped to the chromosome. Genes significantly differentially expressed by the standard approach are labeled with a * next to the gene name. Position of significant fragments is shown as *dotted lines* underneath the genes with their maximum sliding window fold change shown underneath. Stem loop symbols indicate transcriptional terminators. Genes *colored in gray* are part of the operons found to be significantly differentially expressed by the sliding window approach and are drawn to scale. Genes *colored in magenta* are not part of the significant operon and are not drawn to scale. Promoters are indicated by stemmed *arrows*. A window size of 100 nt was used for the smoothing method. Values on the graph represent the center of these 100 nt windows

Traditionally, analysis of RNA-seq data is performed by calculating the normalized RNA-seq coverage (NRC) for a given annotated gene and comparing NRCs between strains with different genetic backgrounds or strains grown under different conditions. When this approach was used here, we initially identified 5 genes that showed significantly higher transcript levels (FDR < 0.05; FC > 2.0) in the *L. monocytogenes* strain overexpressing *sigH (*Δ*BCHL::P*_*rha*_*-sigH)*, as compared to the control strain Δ*BCHL::P*_*rha*_, which does not contain *sigH*. These 5 genes represented LMRG_00908, LMRG_00935 (*comEA*), LMRG_00937 (*comEC*), LMRG_01629 (*lytG*), and LMRG_01643 (*coiA*) (see Additional file 1: Table S1). A total of four σ^H^-dependent promoters were identified upstream of these five genes as *comEA* and *comEC* share the same promoter. Considering these promoters and previously reported operon structures in *L. monocytogenes* [11, 12], these 5 σ^H^-dependent genes were found to represent 2 multigene operons, including (i) LMRG_00908, LMRG_00907 (*dnaG*) and LMRG_00906 (*rpoD*); and (ii) LMRG_00935 (*comEA*), LMRG_00936 (*comEB*) and LMRG_00937 (*comEC*)) and 2 monocistronic genes, *coiA* and *lytG*, for a total of 8 genes that are positively regulated by σ^H^ (Fig.1a–d). We did not find any genes that were significantly downregulated by σ^H^ (FDR < 0.05; FC < 0.5).

Subsequent sliding window analysis, using the same RNA-seq data analyzed with the standard BaySeq approach (described in Methods), identified two additional σ^H^-dependent promoters, in addition to also identifying the promoters described above. The two newly identified promoters, along with previously reported operon structures in *L. monocytogenes* [11, 12], identified two additional multigene operons, including (i) LMRG_00797 to LMRG_00791 and (ii) LMRG_01010 to LMRG_01005) as σ^H^-dependent (Table 1). Overall, the new σ^H^-dependent operons identified using the sliding window approach represent a total of 13 genes that are positively regulated by σ^H^ (Fig. 1e–f). The LMRG_01010-LMRG_01005 operon, which was identified only with the sliding window approach, showed both a σ^H^- and two σ^A^-dependent promoters (Fig. 1f). These data indicate that the sliding window RNA-seq analysis approach described here provides a superior sensitivity for identification of differentially transcribed genes, particularly when multiple promoters are found upstream a gene or operon.

**Table 1:** Results of Sliding Window approach for identification of differentially expressed fragments regulated by σ^H^

Transcriptional Unit	Genes (strand)	Other genes in the same operon	Number of fragments^a^	σ^H^ -dependent promoter (distance from closest gene)	Identified as differentially expressed by standard approach^b^
1	*mpoA* (−)	–	1	No	No
2	*comGG*, *comGF*,*comGB*, *comGA* (−)	*comGC*, *comGD*, *comGE*,	4	Yes (19 nt)	No
3	*rpoD*,*dnaG*, *LMRG_00908* (−)	–	5	Yes (151 nt)	*LMRG_00908*
4	*comEC*,*comEA* (−)	*comEB*	7	Yes (22 nt)	*comEC*, comEA
5	*LMRG_02544* (−)	*LMRG_02542, LMRG_02543, LMRG_02545*	1	No	No
6	*LMRG_01010* (−)	*LMRG_01009*, *LMRG_01008*,*msrA*, *msrB*, *LMRG_01005*	1	Yes (277 nt)	No
7	*coiA* (−)	–	1	Yes (81 nt)	*coiA*
8	*lytG* (−)	–	2	Yes (220 nt)	*lytG*
9	*LMRG_02194* (−)	*LMRG_02196*, *LMRG_02195*, *LMRG_02193*, *LMRG_02192*, *LMRG_02191*, *LMRG_02190*	1	No	No

^a^ Each fragment is composed of one or more 51 nt overlapping windows

^b^This indicates which genes were identified as differentially transcribed with the analysis approach that calculated the normalized RNA-seq coverage for the complete gene (rather than sliding windows) (for full results see Additional file 1: Table S1)

### Newly identified σ^H^-dependent genes include three σ^H^-dependent operons that encode competence proteins

A comparison of the σ^H^-dependent genes identified here with σ^H^-dependent genes and operons that were previously found with microarray based characterization of a *L. monocytogenes* Δ*sigH* strain [9] showed that we identified a number of σ^H^-dependent genes and operons that had not been previously identified, including LMRG1629 (*lytG*), LMRG_00937 (*comEA*), LMRG_00935 (*comEC*), LMRG001643 (*coiA*) (Table 1). The previous microarray study used for comparison used a Δ*sigH* strain in a wildtype background and used a standard probe based microarray (not a tiling array); no additional transcriptomic studies characterizing the σ^H^ regulon in *Listeria* were available for comparison. Interestingly, among the 56 genes identified as directly regulated by σ^H^ in the previous microarray based study of a Δ*sigH* strain [9], only 16 showed a FC >2.0 (the other 40 genes showed FCs between 1.5 and 2.0). Only one of these previously identified genes (LMRG_00908) was also found to be σ^H^-dependent here. This may suggest that a number of genes recognized as σ^H^-dependent in this previous study only show differential σ^H^-dependent transcription in the presence of the other three alternative σ factors, which were not present in the *L. monocytogenes* Δ*BCHL* strain that was used here. These observations further highlight the importance of the use of alternative approaches (e.g., use of a Δ*BCHL* strain overexpressing *sigH*) we have taken here to allow for identification of additional σ^H^-dependent genes. Ectopic and artificial induction of sigma factors and other regulatory proteins has been used successfully in the past to study those regulators where physiological induction signals have not been found [13] and/or where mutants cannot be made [14]. In some cases, large periods of time have passed since linking a set of genes to a regulator (using artificial induction) and discovering a physiological condition where a given regulator and regulated genes are induced. For example in *S. aureus*, σ^H^ dependent transcription of competence genes after overexpression was first reported in 2003 [15], while functional confirmation of σ^H^ contributions to competence were only reported almost 10 years later [16]. While our data reported here thus provide important evidence for contributions of σ^H^ to regulation of competence genes in *L. monocytogenes*, future experiments will be needed to probe the phenotypic importance of this regulatory pathway and to define under which conditions competence gene expression is upregulated by σ^H^ in wildtype *L. monocytogenes*.

Importantly, we identified three σ^H^-dependent transcription units that encode competence proteins, including (i) *comGABCDEFG*, (ii) *comEABC* and (iii) *coiA* (Fig. 1). In *B. subtilis*, a naturally competent bacterium, ComG proteins are required for exogenous DNA to reach the membrane bound receptor ComEA during transformation [17, 18]. Among the genes in the *comEABC* operon, only *comEA* and *comEC* (encoding a polytopic membrane protein that forms the membrane translocation channel) have been shown to be required for transformation in *B. subtilis* [19]. *B. subtilis coiA* has also been shown to be involved in the establishment of DNA transport [20]. Even though competence traditionally implies uptake of DNA, competence genes have been shown to also play roles in survival [21] and virulence [22], processes which do not necessarily require DNA uptake (reviewed in [23]).

In *L. monocytogenes*, competence is not well understood. While this organism seems to possess the machinery for competence, the gene encoding a key regulator of competence in *B. subtilis*, *comK*, is often interrupted by prophages in *L. monocytogenes* strains [24]. Excision of this prophage and restoration of an intact *comK* has been shown to be involved in phagosomal escape and virulence [22] but competence was not tested in this study. The one published study that experimentally tested for competence in *L. monocytogenes* did not find evidence for competence even among two *L. monocytogenes* strains that carried an intact *comK* [25].

Interestingly, *S. aureus* σ^H^ has also been shown to regulate competence-related genes [15]. Although *S. aureus* was originally thought to not be competent, it has now been reported that, facilitated by a complex regulatory mechanism, *S. aureus* cells are able to uptake exogenous DNA, such as antibiotic resistance determinants, through horizontal gene transfer [16]. Even though the specific conditions for competence in *L. monocytogenes* have not been found yet, the fact that this pathogen has conserved σ^H^-dependent mechanisms of regulation of competence genes suggests a specific physiological role of these genes in *L. monocytogenes*.

Among other genes identified as being directly regulated by σ^H^ are the housekeeping genes, *rpoD*, encoding σ^A^ and *dnaG*, encoding the DNA primase (Fig. 1). Another σ^H^-dependent gene, *lytG*, encodes an enzyme with a mannosyl-glycoprotein endo-beta-N-acetylglucosamidase-like domain, which is found in enzymes such as lysozymes and the flagellar protein J that can hydrolyze peptidoglycan [26]. Two genes involved in oxidative stress, *msrA* and *msrB* were also found to be directly regulated by σ^H^, suggesting a possible role of σ^H^ in oxidative stress response [27]. Moreover, the σ^H^-dependent promoter upstream this operon has a long 5’ UTR (270 nt), which overlaps with the 5’ UTR of another gene, *LMRG_01011*, encoded in the opposite strand. *LMRG_01011* encodes a hemolysin III protein (Hly III) and the expression of this protein, could therefore, be σ^H^ regulated post-transcriptionally through RNA interference caused by the 5’ UTR from P_LMRG_01010_.

### σ^H^ - dependent promoters are highly conserved among different *L. monocytogenes* genomes

**Fig. 2:**
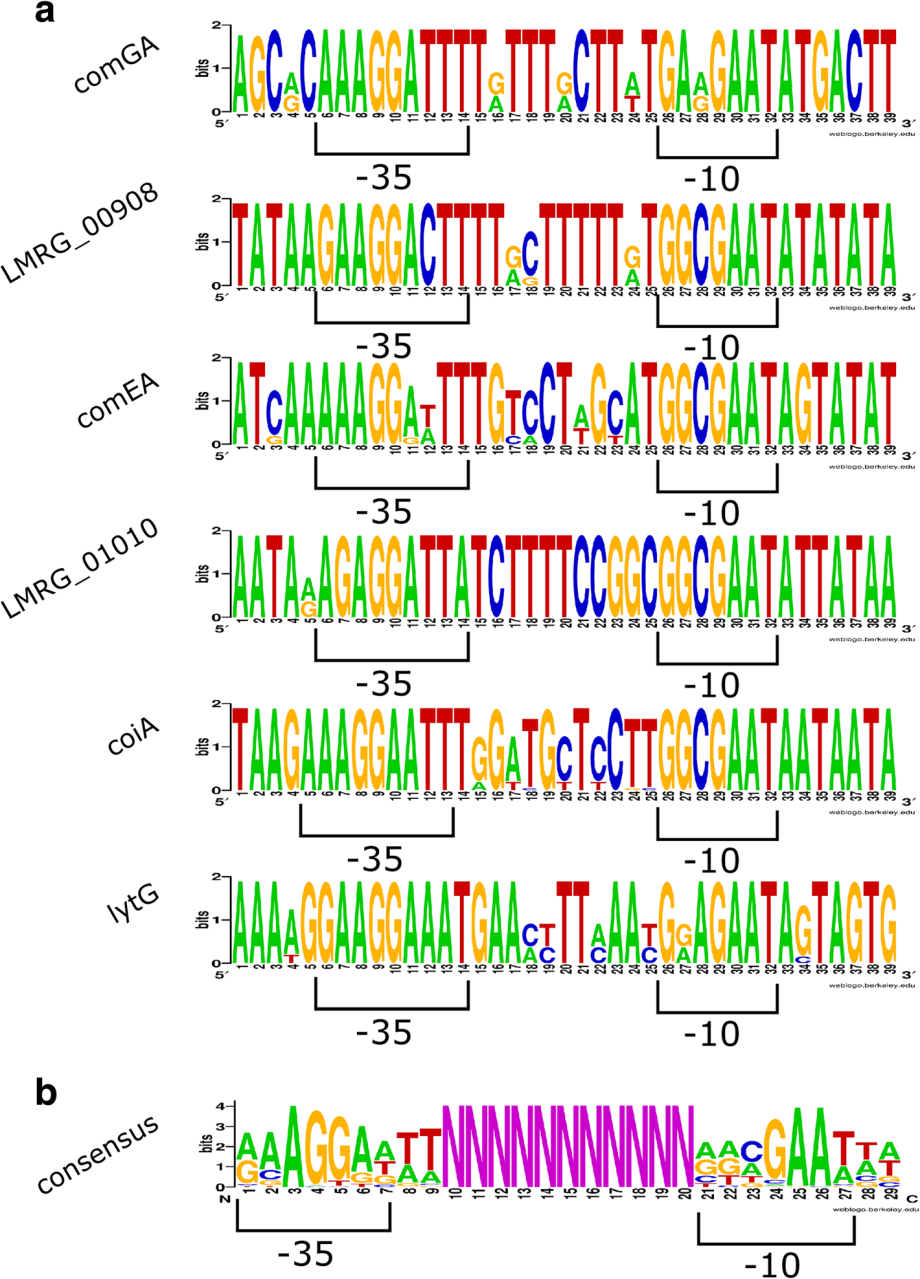
Sequence logos for σ^H^-dependent promoters. **a** Sequence logos for the six σ^H^-dependent promoters found in this study based on alignment of 24 *L. monocytogenes* strains. −35 and −10 regions are shown. **b** Sequence logo of the consensus sequence of σ^H^-dependent promoters based on alignment of sequences from [9]

The six σ^H^-dependent promoters identified in *L. monocytogenes* 10403S were all found to be present in the 23 additional *L. monocytogenes* genomes analyzed here (Additional file 2: Table S2). In general, −35 and −10 promoter regions were highly conserved (Fig. 2, Table 2). For both the *comGA* and the *lytG* promoter sequence, the −35 region was completely conserved across all 24 genomes, while the −10 region presented two variants for each of these two promoters. The *comEA* promoter showed a completely conserved −10 region, while the −35 region showed three variants. On the other hand the −35 region and −10 regions for the three other σ^H^-dependent promoters (upstream of *LMRG_00908, LMRG_01010* and *coiA*) were each completely conserved across all 24 genomes. By comparison, *sigH* was found to be present in all 24 genomes with a high level of conservation (only 4 polymorphic amino acid residues over 201 amino acids). Overall, these data indicate considerable conservation of σ^H^-dependent promoters identified, even though some promoters show lineage or strain specific sequence features. Comparative analysis of the frequency of nucleotide changes within the −35 and −10 signal regions compared to the non-functional sequences (assumed here to be under neutral selection) between these two functional regions suggests that both −35 and −10 signals are under selective pressure among the *L. monocytogenes* strains analyzed (Table 3). This suggests that the σ^H^-dependent promoters identified in this study are functional across all *L. monocytogenes* strains and, therefore, are probably important for *L. monocytogenes* physiology at certain conditions that remain to be established. Future studies are needed to explore the functional importance of the polymorphic sequence features. Interestingly, a previous study [28] found evidence for positive selection of a promoter region that regulates virulence gene expression in *L. monocytogenes*. The sites found under positive selection in this previous study generated a putative σ^B^-dependent promoter in some lineage I strains, which supports that strain and lineage specific promoter region polymorphisms may affect virulence gene expression in *L. monocytogenes*.

**Table 2:** σ^H^ promoter sequences for the σ^H^-dependent operons identified in this study

Promoters	*L. monocytogenes*			*L. innocua*	*L. welshimeri*	*L. ivanovii*	*L. seeligeri*
	Lineage I	Lineage II	Lineage III				
*comGA*							
-35 signal	AAAGGATTT	AAAGGATTT	AAAGGATTT	AAAGGATTT	NF^c^	AAAGGATTT	GAAGGATTT
-10 signal	GAGGAAT	GAAGAAT	GAAGAAT	GTAGAAT	NF	GCAGAAT	GCAGAAT
*LMRG*_*00908*							
-35 signal	GAAGGACTT	GAAGGACTT	GAAGGACTT	GAAGGACTT	GAAGGACTT	GAAGGACTT	GAAGGACTT
-10 signal	GGCGAAT	GGCGAAT	GGCGAAT	GGCGAAT	GTCGAAT	GTCGAAT	GTCGAAT
*comEA*							
-35 signal	AAAGGAATT	AAAGGATTT	AAAGG(G/A)TTT ^a^	AAAGGAATT	NF	NF	AGAGGATTA
-10 signal	GGCGAAT	GGCGAAT	GGCGAAT	GGCGAAT	NF	NF	GGCGAAT
*LMRG*_*01010*							
-35 signal	AGAGGATTA	AGAGGATTA	AGAGGATTA	AGAGGATTA	AGAGGATTA	AGAGGATTA	AGAGGATTA
-10 signal	GGCGAAT	GGCGAAT	GGCGAAT	GGCGAAT	GGCGAAT	CGCGAAT	GGCGAAT
*coiA*							
-35 signal	AAAGGAATT	AAAGGAATT	AAAGGAATT	NF	NF	NF	NF
-10 signal	GGCGAAT	GGCGAAT	GGCGAAT	NF	NF	NF	NF
*lytG*							
-35 signal	GAAGGAAAT	GAAGGAAAT	GAAGGAAAT	NF	NF	NF	NF
-10 signal	G(G/A)AGAAT ^b^	GGAGAAT	GAAGAAT	NF	NF	NF	NF

^a^All *L. monocytogenes* lineage III serotype 4a strains show the sequence AAAGGGTTT, while one lineage III serotype 4c strain shows the sequence AAAGGATTT

^b^Two *L. monocytogenes* lineage I strain (i.e., SLCC2540 and L312) show the sequence GGAGAAT, while all other lineage I strains show the sequence GAAGAAT

^c^ NF: Not found

**Table 3:** Conservation of identified σ^H^-dependent promoters across *Listeria monocytogenes* genomes

Gene	N° of polymorphic sites within −35 and −10 signal (out of 16 nt total)	N° of polymorphic sites between −35 and −10 signal (out of 11 nt total)	Probability^a^
*comGA*	1 (6 %)	3 (27 %)	0.0084
*LMRG_00908*	0 (0 %)	3 (27 %)	0.0061
*comEA*	2 (13 %)	4 (36 %)	0.0014
*LMRG_01010*	0 (0.00 %)	0 (0 %)	1.000
*coiA*	0 (0.00 %)	4 (36 %)	0.0007
*lytG*	1 (6 %)	4 (36 %)	0.0011

^a^Probability of finding only as many polymorphic sites in the −35 and −10 sequences combined given the polymorphism frequency in between −35 and −10 signals for the same promoter; a significant probability (<0.05) provides evidence for higher conservation of the promoter regions than expected based on the conservation observed for the region between the −10 and the −35 region

### σ^H^ promoters and the σ^H^ regulon have diverged among different *Listeria* species

Further analysis of the six σ^H^-dependent promoters identified in *L. monocytogenes* showed that only the *LMRG*_*00908* and *LMRG*_*01010* promoters were found in all 5 *Listeria* species analyzed (i.e., *L. monocytogenes*, *L. innocua*, *L. ivanovii*, *L. welshimeri* and *L. seeligeri*). The *comGA* promoter was not found in the *L. welshimeri* genome (Additional file 2: Table S2). The *comEA* promoter was not found in the *L. ivanovii* and *L. welshimeri* genomes and the *coiA* and *lytG* promoters were only found in the *L. monocytogenes* genomes. Analyses of the genes transcribed by these promoters revealed the respective genes were also missing in the genomes where a promoter could not be found. Therefore, the competence genes represented by the *comGABCDEFG* operon, the *comEABC* operon and the *coiA* gene are not found across all *Listeria* species and the σ^H^ regulon is not conserved across these species. Taking into consideration that a *sigH* mutant has been shown to have reduced virulence in a mouse model [8], the absence of these σ^H^-dependent genes in non-pathogenic *Listeria* spp. could indicate that these genes may have evolved to contribute σ^H^-dependent virulence related functions in *L. monocytogenes*. By comparison, *sigH* was present in all *Listeria* species genomes; in addition to the 4 polymorphic amino acid residues found in *L. monocytogenes*, a further 18 polymorphic amino acid residues were found in the *Listeria* spp. genomes.

**Fig. 3:**
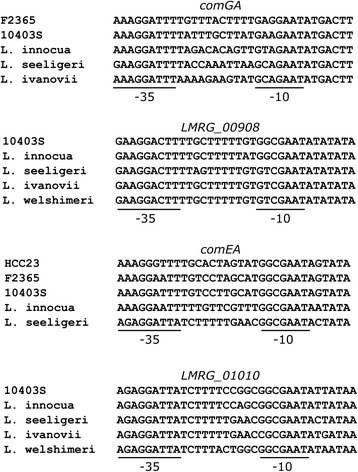
Alignment of σ^H^-dependent promoters found in *Listeria* species. *L. monocytogenes* strains 10403S (lineage II), F2365 (lineage I) and HCC23 (lineage III) are used for comparison. −35 and −10 regions are shown

Among the promoters found in other *Listeria* species besides *L. monocytogenes*, only the *LMRG_00908* operon was highly conserved (Fig. 3). This promoter showed a completely conserved −35 region, while two variants of the −10 region were found. The *comEA* promoter, which was only found in *L. monocytogenes*, *L. innocua* and *L. seeligeri*, had a perfectly conserved −10 region across all three species. However, besides the two variants of the −35 region found among the *L. monocytogenes* strains, each the *L. innocua* and the *L. seeligeri* strain presented distinct −35 sequences. The *comGA* promoter showed variable −35 and −10 regions across the *Listeria* species. The *LMRG_01010* -35 sequence in *L. innocua*, *L. ivanovii*, *L. welshimeri* and *L. seeligeri* matched that of the *L. monocytogenes* strains. The only −10 region that diverged from the others was that found in the *L. ivanovii* strain. Therefore, in addition to a σ^H^ regulon that is not conserved across all *Listeria* species, there is also some variation in the σ^H^-dependent promoters that are present in other *Listeria* species. While the σ^H^-dependent promoters present generally seem to be conserved enough to suggest conservation of σ^H^-dependent regulation, future experimental studies are needed to explore the potential functional importance of these polymorphic sequence features.

## Conclusions

Combined with prior data that indicated a role of σ^H^ in virulence in a mouse model, identification of a number of σ^H^-dependent *L. monocytogenes* competence genes that are conserved in the pathogen *L. monocytogenes*, but not in other non-pathogenic *Listeria* strains, suggests a possible novel role of σ^H^-dependent competence genes in *L. monocytogenes* virulence. The development and use of a sliding window approach to identify differential transcription using RNA-seq data not only allowed for identification of new σ^H^-dependent promoters in *L. monocytogenes*, but also provides a general approach for sensitive identification of differentially transcribed promoters and genes. We predict that this approach will be particularly valuable for identification of differentially transcribed genes and genomic regions that are transcribed from multiple and possibly redundant promoter elements.

## Methods

### Bacterial strains, mutant construction, and growth conditions

The quadruple alternative σ factor mutant *(*Δ*BCHL*; FSL C3-135*)* of *L. monocytogenes* strain 10403S [29] was used as the background strain in this study. This strain was modified to overexpress *sigH* from a rhamnose inducible promoter. Briefly, the *sigH* gene was amplified from *L. monocytogenes* 10403S by PCR and cloned into the plasmid pLF1 [30] downstream of the rhamnose inducible promoter P_*rha*_. The plasmid construct was confirmed by PCR and sequencing. All the cloning steps were performed in *E. coli* DH5α (NEB). The final plasmid was transformed into *E. coli* strain SM10 to allow for conjugation of the plasmid into *L. monocytogenes* 10403S Δ*BCHL*, followed by chromosomal integration of the P_*rha*_*sigH* construct (yielding strain 10403S::Δ*BCHL P*_*rha*_*-sigH*; FSL B2-426). A control strain (Δ*BCHL-P*_*rha*_; FSL B2-429) was constructed by introducing the empty plasmid pLF1 into the chromosome of *L. monocytogenes* 10403S Δ*BCHL* through conjugation and chromosomal integration. Transconjugants were selected with 200 μg/ml Streptomycin and 7.5 μg/ml Chloramphenicol, and confirmed by PCR.

For RNA isolation, strains were streaked from frozen Brain Heart Infusion (BHI) stock, stored at −80 °C in 15 % glycerol, onto a BHI agar plate, followed by incubation at 37 °C for 24 h. A single colony was subsequently inoculated into 5 ml of BHI broth in 16 mm tubes, followed by incubation at 37 °C with shaking (230 rpm) for 18 h (Series 25 Incubator, New Brunswick Scientific, Edison, NJ). After 18 h, 50 μl BHI culture was inoculated into fresh 5 ml BHI broth and grown to OD_600_ 0.4–0.5 at 37 °C.

### Rhamnose induction

Induction of *sigH* transcription was performed by adding 250 μl of 1M rhamnose stock solution to 5 ml OD_600_ 0.4–0.5 bacterial cultures (for a final concentration of 50 mM rhamnose), followed by incubation at 37 °C for an additional 30 min. Induction with rhamnose was performed for both 10403S::*ΔBCHL P*_*rha*_*-sigH* and Δ*BCHL-P*_*rha*_. qRT-PCR using the SYBR Green Master Mix Reagent (Life Technologies) and the ABI Prism 7000 Sequence Detection System (Applied Biosystems, Foster City, CA) determined that the optimum rhamnose concentration for *sigH* induction was 50 mM rhamnose. Transcript levels were determined for *sigH* and the housekeeping gene *rpoB* in strain 10403S::Δ*BCHL P*_*rha*_*-sigH*. Expression level differences were determined by ΔΔCt method [31] using the housekeeping gene *rpoB* as reference gene.

### RNA isolation

RNA isolation was performed as previously described by our group [32]; minor modifications were made to this previous RNA isolation protocol. Briefly, for each sample, 3 ml of RNAprotect bacteria reagent (Qiagen, Valencia, CA) was added to 3 ml of bacterial culture. The mix was incubated at room temperature for 10 min to ensure that the bacterial RNA was stabilized. Cells were pelleted by centrifugation (4,637 × *g*, 30 min) at 4 °C and suspended in nuclease free water with proteinase K (25 mg/ml) and lysozyme (50 mg/ml), followed by incubation at 37 °C for 30 min. After RNA isolation with TRI reagent, total RNA was incubated with Turbo DNase (Life Technologies) to remove remaining DNA in the presence of RNasin (Promega). Subsequently, RNA was purified using phenol-chloroform/chloroform extractions, followed by precipitation and resuspension. Purity of RNA and efficiency of the DNase treatment was assessed by UV spectrophotometry (Nanodrop, Wilmington, DE) and qRT-PCR for the housekeeping gene *rpoB* (all samples showed Ct > 35, indicating absence of DNA contamination at relevant levels), respectively. All experiments were performed in three biological replicas.

### cDNA libraries and RNA-seq

Preparation of directional cDNA fragment libraries was performed using the ScriptSeq Complete Kit (Bacteria)-Low Input (Epicentre, Madison, WI). 16S and 23S rRNA was removed from total RNA with Ribo-Zero rRNA Removal Reagents (Bacteria)-Low Input and Magnetic Core Kit-Low Input. rRNA-depleted samples were run on the 2100 Bioanalyzer (Agilent Technology, Santa Clara, CA) to confirm reduction of 16S and 23S rRNA and followed by purification using Agencourt RNAClean XP Kit (Beckman Coulter Inc, Brea, CA). Indexed RNA-seq libraries were quantified by digital PCR and sequencing was carried out on a Hiseq 2500 (single-end, 150-bp per read) at the Cornell Core Facility for RNA-sequencing.

### RNA-Seq alignment, coverage and differential expression analysis

Sequence reads were aligned to a 10403S genome using the BWA mem algorithm in BWA version 0.7.3a [33] and the data for coverage per base on sense and antisense strands were analyzed separately using samtools [34].

Differential expression of genes in the two different strains (Δ*BCHL::P*_*rha*_ and Δ*BCHL::P*_*rha-*_*sigH)* was initially analyzed using the Bayseq package for R version 2.2.0 [35]. Genes were considered differentially expressed if the FDR (False Discovery Rate) was < 0.05 and the FC (Fold Change) was > 2.0 or < 0.5 (FC = average (Δ*BCHL-P*_*rha-*_*sigH)***/**average (Δ*BCHL-P*_*rha*_)).

### Promoter search using a Sliding Window approach

In order to identify further σ^H^-dependent promoters that may not result in differential expression of the actual gene due to the coexistence of σ^A^-dependent promoters regulating a given gene, a sliding window approach was implemented. Three different window sizes and window sliding values were used (window sizes = 26, 51 and 102 nt; window sliding values = 13, 25 and 50 nt, respectively), which all resulted in the identification of the same promoters. However, the combination of window size = 51 nt and window sliding = 25 nt resulted in a lower number of fragments that were not preceded by upstream promoters (which was considered a “false positive” finding); a sliding window size of 51 yielded 3 false positive fragments as compared to 18 and 10 with sliding windows sizes of 25 and 100, respectively. Therefore, results obtained with window size = 51 nt and window sliding = 25 nt are presented here. Briefly, the 10403S genome was divided into windows of 51 nt (window size) with 25 nt overlap (window sliding) and the RNA-Seq coverage was obtained for each of the 116,123 resulting windows. RNA-Seq coverage was obtained as described above and the coverage per nt was used to obtain the total coverage of each window (e.g., the coverage of the window ranging from nt 1 to nt 51 is the sum of the coverage of each nt between nt 1 and nt 51). Bayseq version 2.2.0 was then used, as described above, to identify windows with significant differential expression between the Δ*BCHL::P*_*rha-*_*sigH* strain and the Δ*BCHL::P*_*rha*_ strain. Fold change was also calculated as described above and windows presenting an FDR < 0.05 and FC > 2.0 were considered for further analysis. Windows matching the required thresholds were then sorted and overlapping windows were considered part of the same fragment. Fragments that were mapped to the same gene or operon were considered as being part of the same transcriptional unit (TU). The 5’ end of significant TUs were then manually scanned for promoter sequences located 5 to 30 nt upstream the identified transcriptional start sites using the genome browser Artemis to identify σ^H^-dependent promoters based on the consensus sequence (A/G)(A/C)AGG(A/G)(A/T)(A/T)(A/T) – N_11-12nt_ – (A/G)(A/G)(A/C)GAA(A/T)) [9] (Additional file 3: Table S3).

### Identification and characterization of σ^H^-dependent promoters in additional *L. monocytogenes* and *Listeria* spp. genomes

A total of 28 finished genome sequences representing 10 *L. monocytogenes* lineage I strains, 10 *L. monocytogenes* lineage II strains (including 10403S), 4 *L. monocytogenes* lineage III strains, 1 *L. innocua* strain, 1 *L. ivanovii* strain, 1 *L. welshimeri* strain and 1 *L. seeligeri* strain were retrieved from NCBI GenBank database (Additional file 2: Table S2). Standalone Blast searches using the σ^H^-dependent promoter sequences or their respective regulated genes as queries were carried out against two databases containing (i) all 24 *L. monocytogenes* genomes (*L. monocytogenes* database) and (ii) all 4 non-*L. monocytogenes* genomes (*Listeria* spp. database). Blast searches against both databases were carried out with the following parameters: “Expectation Value (E)” set to 0.2, “Word Size” set to 8, and “Filter Query Sequence (DUST)” set to FALSE. For searches against the *L. monocytogenes* database, the “Penalty for Nucleotide Mismatch” was set to −4 while the “Reward for a Nucleotide Match” was set to 5. For searches against the *Listeria* spp. database, the mismatch penalty was set to −2 and the reward for a match was set to 2 to allow for matches against more divergent sequences. Matches against the query sequences were parsed from the output using in-house Perl scripts. Only the best match against each query was retrieved and sequences were aligned using standalone ClustaW [36]. Two or more equally best matches were not obtained in any of the BLAST searches. BLAST results and alignments are available and will be provided by request. Sequence logos were created using the WebLogo generator available online at http://weblogo.berkeley.edu/logo.cgi [37].

### Availability of supporting data

RNA-seq data have been deposited in NCBI's Gene Expression Omnibus and are accessible through GEO Series accession number GSE73008 (https://www.ncbi.nlm.nih.gov/geo/query/acc.cgi?acc=GSE73008).

## Additional files

Additional file 1: Table S1. Genes identified as differentially expressed between *L. monocytogenes* overexpressing *sigH* (10403S::Δ*sigBCHL P*_*rha*_ *-sigH*) and a Δ*sigBCHL* control strain, based on the RNA-seq coverage data calculated for complete ORFs. (DOCX 15 kb) Additional file 2: Table S2. Listeria genomes used for comparative analysis of SigmaH-dependent promoters. (XLSX 10 kb) Additional file 3: Table S3. Significant fragments found with the sliding window approach. (XLSX 25 kb)

## Abbreviations

BHI: Brain Heart Infusion
BWA: Burrows-Wheeler Aligner
FC: fold change
FDR: False Discovery Rate
NEB: New England Biolabs
NRC: normalized RNA-seq coverage
ORF: open reading frame
RNA-seq: RNA sequencing
TUs: transcription units
UTR: untranslated region

## References

[1] Feklistov A, Sharon BD, Darst SA, Gross CA (2014). Bacterial sigma factors: a historical, structural, and genomic perspective. Annu Rev Microbiol.

[2] Mujahid S, Orsi RH, Vangay P, Boor KJ, Wiedmann M (2013). Refinement of the *Listeria monocytogenes* sigmaB regulon through quantitative proteomic analysis. Microbiology.

[3] Kazmierczak MJ, Mithoe SC, Boor KJ, Wiedmann M (2003). *Listeria monocytogenes* sigma B regulates stress response and virulence functions. J Bacteriol.

[4] Palmer ME, Chaturongakul S, Wiedmann M, Boor KJ. The *Listeria monocytogenes* sigmaB regulon and its virulence-associated functions are inhibited by a small molecule. MBio. 2011;2(6). doi: 10.1128/mBio.00241-11. 10.1128/mBio.00241-11 PMC3225968 22128349

[5] Grossman AD (1995). Genetic networks controlling the initiation of sporulation and the development of genetic competence in *Bacillus subtilis*. Annu Rev Genet.

[6] Britton RA, Eichenberger P, Gonzalez-Pastor JE, Fawcett P, Monson R, Losick R, Grossman AD (2002). Genome-wide analysis of the stationary-phase sigma factor (sigma-H) regulon of *Bacillus subtilis*. J Bacteriol.

[7] Chaturongakul S, Raengpradub S, Wiedmann M, Boor KJ (2008). Modulation of stress and virulence in *Listeria monocytogenes*. Trends Microbiol.

[8] Rea RB, Gahan CG, Hill C (2004). Disruption of putative regulatory loci in *Listeria monocytogenes* demonstrates a significant role for Fur and PerR in virulence. Infect Immun.

[9] Chaturongakul S, Raengpradub S, Palmer ME, Bergholz TM, Orsi RH, Hu Y, Ollinger J, Wiedmann M, Boor KJ (2011). Transcriptomic and phenotypic analyses identify coregulated, overlapping regulons among PrfA, CtsR, HrcA, and the alternative sigma factors sigmaB, sigmaC, sigmaH, and sigmaL in *Listeria monocytogenes*. Appl Environ Microbiol.

[10] Shell SS, Wang J, Lapierre P, Mir M, Chase MR, Pyle MM, Gawande R, Ahmad R, Sarracino DA, Ioerger TR et al. Leaderless transcripts and small proteins are common features of the mycobacterial translational landscape. PLoS Genet. 2015;11(11):e1005641. 10.1371/journal.pgen.1005641 PMC4633059 26536359

[11] Toledo-Arana A, Dussurget O, Nikitas G, Sesto N, Guet-Revillet H, Balestrino D, Loh E, Gripenland J, Tiensuu T, Vaitkevicius K et al. The *Listeria* transcriptional landscape from saprophytism to virulence. Nature. 2009;459(7249):950–6. 10.1038/nature08080 19448609

[12] Oliver HF, Orsi RH, Ponnala L, Keich U, Wang W, Sun Q, Cartinhour SW, Filiatrault MJ, Wiedmann M, Boor KJ. Deep RNA sequencing of *L. monocytogenes* reveals overlapping and extensive stationary phase and sigma B-dependent transcriptomes, including multiple highly transcribed noncoding RNAs. BMC Genomics. 2009;10:641. 10.1186/1471-2164-10-641 PMC2813243 20042087

[13] Taniguchi H, Wendisch VF (2015). Exploring the role of sigma factor gene expression on production by *Corynebacterium glutamicum*: sigma factor H and FMN as example. Front Microbiol.

[14] Leang C, Krushkal J, Ueki T, Puljic M, Sun J, Juarez K, Nunez C, Reguera G, DiDonato R, Postier B et al. Genome-wide analysis of the RpoN regulon in Geobacter sulfurreducens. BMC Genomics. 2009;10:331. 10.1186/1471-2164-10-331 PMC2725144 19624843

[15] Morikawa K, Inose Y, Okamura H, Maruyama A, Hayashi H, Takeyasu K, Ohta T. A new staphylococcal sigma factor in the conserved gene cassette: functional significance and implication for the evolutionary processes. Genes Cells. 2003;8(8):699–712. 10.1046/j.1365-2443.2003.00668.x 12875655

[16] Morikawa K, Takemura AJ, Inose Y, Tsai M, Nguyen Thi le T, Ohta T, Msadek T. Expression of a cryptic secondary sigma factor gene unveils natural competence for DNA transformation in *Staphylococcus aureus*. PLoS Pathog. 2012;8(11):e1003003. 10.1371/journal.ppat.1003003 PMC3486894 23133387

[17] Chung YS, Breidt F, Dubnau D (1998). Cell surface localization and processing of the ComG proteins, required for DNA binding during transformation of *Bacillus subtilis*. Mol Microbiol.

[18] Chung YS, Dubnau D (1998). All seven comG open reading frames are required for DNA binding during transformation of competent *Bacillus subtilis*. J Bacteriol.

[19] Provvedi R, Dubnau D (1999). ComEA is a DNA receptor for transformation of competent *Bacillus subtilis*. Mol Microbiol.

[20] Kramer N, Hahn J, Dubnau D (2007). Multiple interactions among the competence proteins of *Bacillus subtilis*. Mol Microbiol.

[21] Charpentier X, Polard P, Claverys JP (2012). Induction of competence for genetic transformation by antibiotics: convergent evolution of stress responses in distant bacterial species lacking SOS?. Curr Opin Microbiol.

[22] Rabinovich L, Sigal N, Borovok I, Nir-Paz R, Herskovits AA (2012). Prophage excision activates *Listeria* competence genes that promote phagosomal escape and virulence. Cell.

[23] Johnston C, Martin B, Fichant G, Polard P, Claverys JP (2014). Bacterial transformation: distribution, shared mechanisms and divergent control. Nat Rev Microbiol.

[24] Loessner MJ, Inman RB, Lauer P, Calendar R (2000). Complete nucleotide sequence, molecular analysis and genome structure of bacteriophage A118 of *Listeria monocytogenes*: implications for phage evolution. Mol Microbiol.

[25] Borezee E, Msadek T, Durant L, Berche P (2000). Identification in Listeria monocytogenes of MecA, a homologue of the *Bacillus subtilis* competence regulatory protein. J Bacteriol.

[26] Nambu T, Minamino T, Macnab RM, Kutsukake K (1999). Peptidoglycan-hydrolyzing activity of the FlgJ protein, essential for flagellar rod formation in *Salmonella typhimurium*. J Bacteriol.

[27] Singh VK, Vaish M, Johansson TR, Baum KR, Ring RP, Singh S, Shukla SK, Moskovitz J. Significance of four methionine sulfoxide reductases in *Staphylococcus aureus*. PLoS One. 2015;10(2):e0117594. 10.1371/journal.pone.0117594 PMC4334518 25680075

[28] Orsi RH, Maron SB, Nightingale KK, Jerome M, Tabor H, Wiedmann M (2008). Lineage specific recombination and positive selection in coding and intragenic regions contributed to evolution of the main *Listeria monocytogenes* virulence gene cluster. Infect Genet Evol.

[29] Mujahid S, Orsi RH, Boor KJ, Wiedmann M (2013). Protein level identification of the *Listeria monocytogenes* sigma H, sigma L, and sigma C regulons. BMC Microbiol.

[30] Fieseler L, Schmitter S, Teiserskas J, Loessner MJ (2012). Rhamnose-inducible gene expression in *Listeria monocytogenes*. PLoS One.

[31] Livak KJ, Schmittgen TD (2001). Analysis of relative gene expression data using real-time quantitative PCR and the 2(−Delta Delta C(T)) Method. Methods.

[32] Tang S, Orsi RH, den Bakker HC, Wiedmann M, Boor KJ, Bergholz TM (2015). Transcriptomic analysis of the adaptation of *Listeria monocytogenes* to growth on vacuum-packed cold smoked salmon. Appl Environ Microbiol.

[33] Li H, Durbin R (2010). Fast and accurate long-read alignment with Burrows-Wheeler transform. Bioinformatics.

[34] Li H, Handsaker B, Wysoker A, Fennell T, Ruan J, Homer N, Marth G, Abecasis G, Durbin R, Genome Project Data Processing S. The Sequence Alignment/Map format and SAMtools. Bioinformatics. 2009;25(16):2078–9. 10.1093/bioinformatics/btp352 PMC2723002 19505943

[35] Hardcastle TJ, Kelly KA (2010). baySeq: empirical Bayesian methods for identifying differential expression in sequence count data. BMC Bioinformatics.

[36] Larkin MA, Blackshields G, Brown NP, Chenna R, McGettigan PA, McWilliam H, Valentin F, Wallace IM, Wilm A, Lopez R et al. Clustal W and Clustal X version 2.0. Bioinformatics. 2007;23(21):2947–8. 10.1093/bioinformatics/btm404 17846036

[37] Crooks GE, Hon G, Chandonia JM, Brenner SE (2004). WebLogo: a sequence logo generator. Genome Res.

