# Supplementary material for: An advanced bioinformatics approach for analyzing RNA-seq data reveals sigma H-dependent regulation of competence genes in Listeria monocytogenes
Source: BMC Genomics. 2016 Feb 16;17:115. doi: 10.1186/s12864-016-2432-9 (PMC4754846; doi:10.1186/s12864-016-2432-9)
Supplement: Additional file 1: Table S1. — Genes identified as differentially expressed between L. monocytogenes overexpressing sigH (10403S::ΔsigBCHL P rha -sigH) and a ΔsigBCHL control strain, based on the RNA-seq coverage data calculated for complete ORFs. (DOCX 15 kb) [file 12864_2016_2432_MOESM1_ESM.docx]

**Additional file 1: Table S1.** Genes identified as differentially expressed between *L. monocytogenes* overexpressing *sigH* (10403S::Δ*sigBCHL P_rha_-sigH*) and a Δ*sigBCHL* control strain, based on the RNA-seq coverage data calculated for complete ORFs

| Gene Name in | | | Gene Product in 10403S | | Average NRC^a^ for | | | FC | | FDR | |
| --- | --- | --- | --- | --- | --- | --- | --- | --- | --- | --- | --- |
| 10403S | EGD-e |  | | Prha-sigH | | Prha |  | |  | |  |
| LMRG_01629 | lmo2203 (lytG) | N-acetylmuramoyl-L-alanine amidase, family 4 (autolysin) | | 48,503.00 | | 2,972.33 | 16.32 | | 2.2E-04 | |  |
| LMRG_00937 | lmo1484(*comEA*) | Late competence protein ComEA, DNA receptor | | 46,724.67 | | 1,307.67 | 35.73 | | 6.6E-03 | |  |
| LMRG_00908 | lmo1456 | Hypothetical protein | | 548,170.67 | | 137,001.33 | 4.00 | | 1.7E-02 | |  |
| LMRG_00935 | lmo1482(*comEC*) | Late competence protein ComEC, DNA transport | | 136,101.00 | | 63,038.00 | 2.16 | | 3.5E-02 | |  |
| LMRG_01643 | lmo2189(*coiA*) | Competence protein CoiA | | 8,6365.33 | | 29,506.00 | 2.93 | | 4.8E-02 | |  |

^a.^NRC normalized RNA-seq coverage
